# RECURRENT OVARIAN MALIGNANCY PRESENTING AS CUTANEOUS METASTASIS

**DOI:** 10.4103/0019-5154.57620

**Published:** 2009

**Authors:** Shilpaja J Karpate, Swarnlata L Samal, Suchi M Jain

**Affiliations:** *From the Department of Obstetrics and Gynecology, Mahatma Gandhi Institute of Medical Sciences, Sewagram, Wardha, Maharashtra, India.*

**Keywords:** *Skin metastasis*, *ovarian carcinoma*, *prognosis*

## Abstract

Cutaneous metastasis from ovarian carcinoma is relatively uncommon in clinical practice. We report the case of the woman who presented to us with clitoral nodules and skin nodules. Histopathological examination of nodules confirmed the diagnosis of metastasis of an ovarian carcinoma. Despite poor prognosis, the patient responded and survived well beyond the expected four months survival of similar cases.

## Introduction

The skin is a relatively uncommon site for distant metastatic deposits compared with organs such as liver, lungs and bones. Almost nine percent of internal cancers may have skin metastases and in about 0.5-1%, it is the presenting feature. The most common sources of cutaneous metastases are breast, lungs, colon, stomach, upper aerodigestive tract, uterus, and kidney;[1] while only four percent are from the ovaries. Skin metastases from ovarian carcinoma is rarely reported. Most cases who present as cutaneous nodules have periumbilical Sister Joseph's nodules. A distressing case of epithelial ovarian carcinoma complicated by groin lymph node metastatic disease and peau d'orange edema with multiple skin nodules is reported here.

## Case Report

A 52 years woman, para five, presented with pain in the lower abdomen, swelling of both the lower limbs and nodular eruptions on the lower abdomen, buttocks and thighs over three months. She attained menopause ten years back. She had undergone primary surgery at a private hospital for a left ovarian mass two years back, which was then removed. She reported to the same doctor with enlarged supraclavicular left lymph node after three months, fine needle aspiration cytology of which was reported as metastatic adenocarcinoma. She was commenced on chemotherapy; six cycles of cisplatin and cyclophosphamide were given. The doses of which were not known to us. She subsequently underwent second laparotomy for a right ovarian mass of 8 × 8 cm during which the right ovarian mass was removed and omentectomy was done. Histology was reported as a serous cystadenocarcinoma grade 3 with capsular invasion of right ovary with no omental deposits or peritoneal spread. She was then discharged by her doctor.

**Figure 1:**
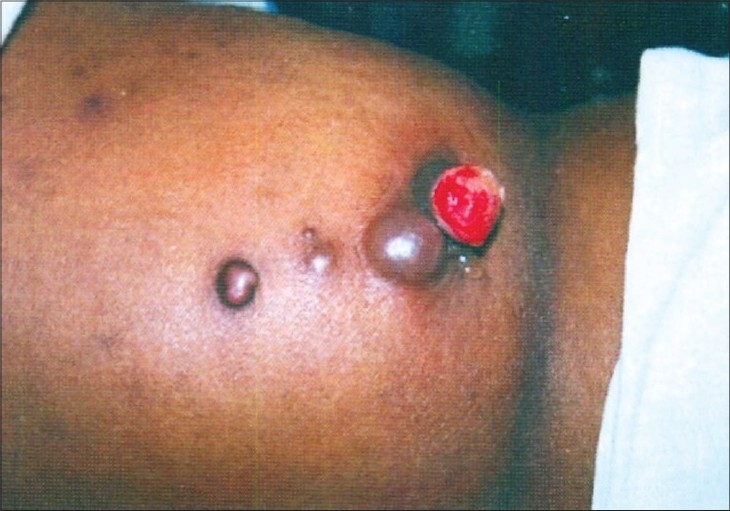
Metastatic skin nodules as seen on the left buttock

When she presented to us, she had palpable lymph nodes in the left axilla and bilateral superficial inguinal lymph nodes, which were hard, non tender, and fixed. There was bilateral nonpitting edema and multiple nodular swellings on both lower limbs. The skin was fixed to the nodules on the left buttock and left thigh. The largest nodule was of size 4 × 2 cm and the skin over it was eroded with sero-sanguineous discharge [Figure 1]. On abdominal examination, two scars of previous laparotomy were present. On local examination, two nodular swellings were present on the clitoris of size 0.5 cm × 5 cm. On speculum examination, it was found that the cervix was flush with the vagina and on bimanual examination, the uterine size could not be made out.

**Figure 2:**
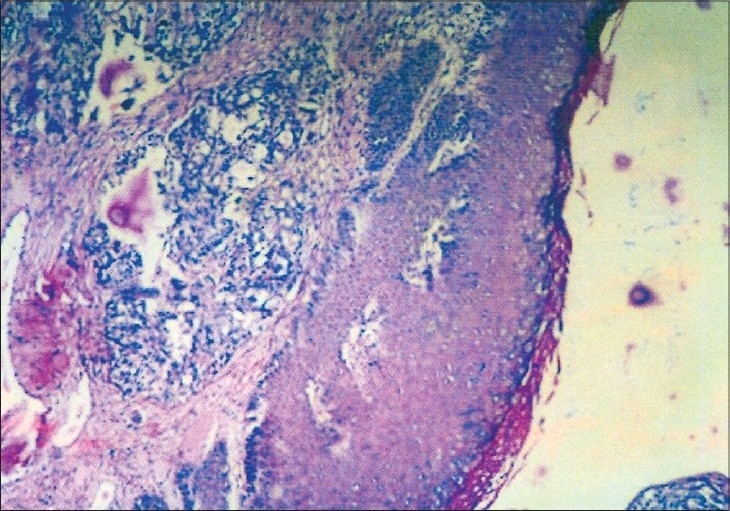
Microphotograph shows the presence of metastatic adenocarcinoma beneath the normal epidermis(H and E,×200)

Excision biopsy of clitoral nodules and fine needle aspiration cytology of skin nodules were reported as metastatic adenocarcinoma. The clitoral nodules showed sub-epithelial and overlying epidermal involvement [Figure 2]. Fine needle aspiration cytology of inguinal lymph nodes was also suggestive of metastatic adenocarcinoma. The CA125 levels were 1438.38 mIU/l.

In view of the above findings which correlated with recurrent malignancy, she was put on palliative combination chemotherapy comprising of Paclitaxel (150 mg/m^2^), Cisplatin (50 mg/m^2^) and Doxurubicin (60 mg/m2) for six cycles. She had received six cycles of combination chemotherapy, each cycle starting 21 days from the last. The skin nodules have regressed in size, skin over nodule has healed, and lymphoedema has disappeared. Her CA125 levels have also come down to 25 mIU/l.

## Discussion

Cutaneous metastases generally present as solitary or multiple, painless, firm to hard nodules, which may be skin colored, blue brown, reddish purple, or morphea-like sclerotic form. The sites for affection are skin in the vicinity of affected organ, umbilicus (Sister Mary Joseph's nodule) and recent operative scars. Cutaneous metastasis may be mistaken for a cyst or an inflammatory lesion.[2] Metastasis to the skin occurs as a result of lymphatic or hematogenous dissemination of tumor. The tumor cells may be identifiable as having a specific organ of origin or may be anaplastic. Generally, they resemble the cells of primary tumor. Cutaneous disease is usually indicative of a poor prognosis. Patients with solitary metastasis without other evidence of dissemination may have a better survival. Treatment options depend on the primary tumor and include excision or other destructive therapy for limited number of lesions (laser, radiotherapy, photodynamic) and chemotherapy or other systemic therapy for disseminated lesions.

A report by Eckman *et al.,*[3] describes two cases of metastases from ovarian tumours and stresses that through recognition of appearance and distribution of the lesions, the physician may suspect the diagnosis. Skin involvement is a late complication that occurs rarely in ovarian cancer patients. Prognosis after skin metastases is poor. A study by Cormio *et al.,*[4] describes nine cases of skin metastases of ovarian carcinoma. They found that it occurs 23.4 + 12 months after the diagnosis of ovarian cancer. The diameter of lesion ranged from 0.5 cm to 3 cm. Median survival after diagnosis was four months. Our case is unusual because distant metastasis presented early in disease and in absence of local spread to coelom. It probably represents multifocal origin of tumor. Epidermis is usually spared in metastatic lesion, but here the overlying epidermis was also involved. The other unique feature of this case was the large size of the nodules and their response to multi-drug chemotherapy with regression of metastatic changes.

Thus, in conclusion, it can be said that despite such a rare complication and poor prognosis, the patient responded well to the palliative treatment. Further study is needed to be undertaken for confirming the perfect treatment regimen.
